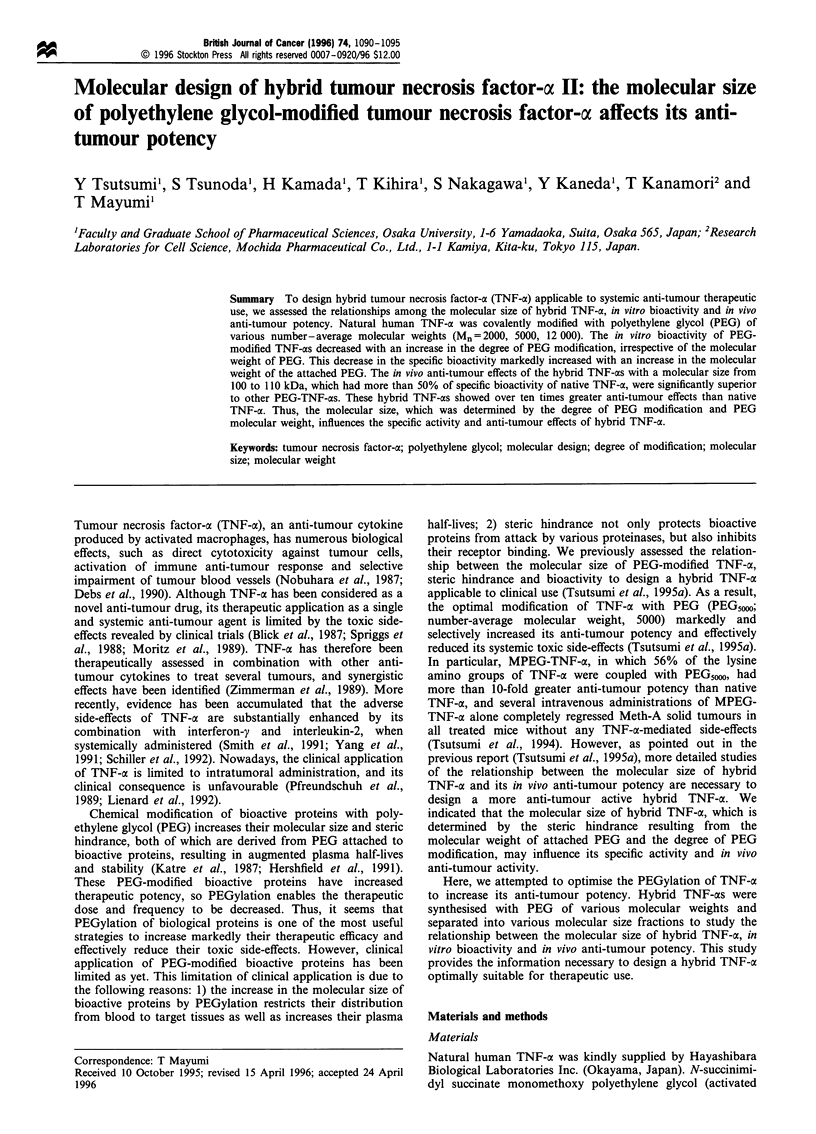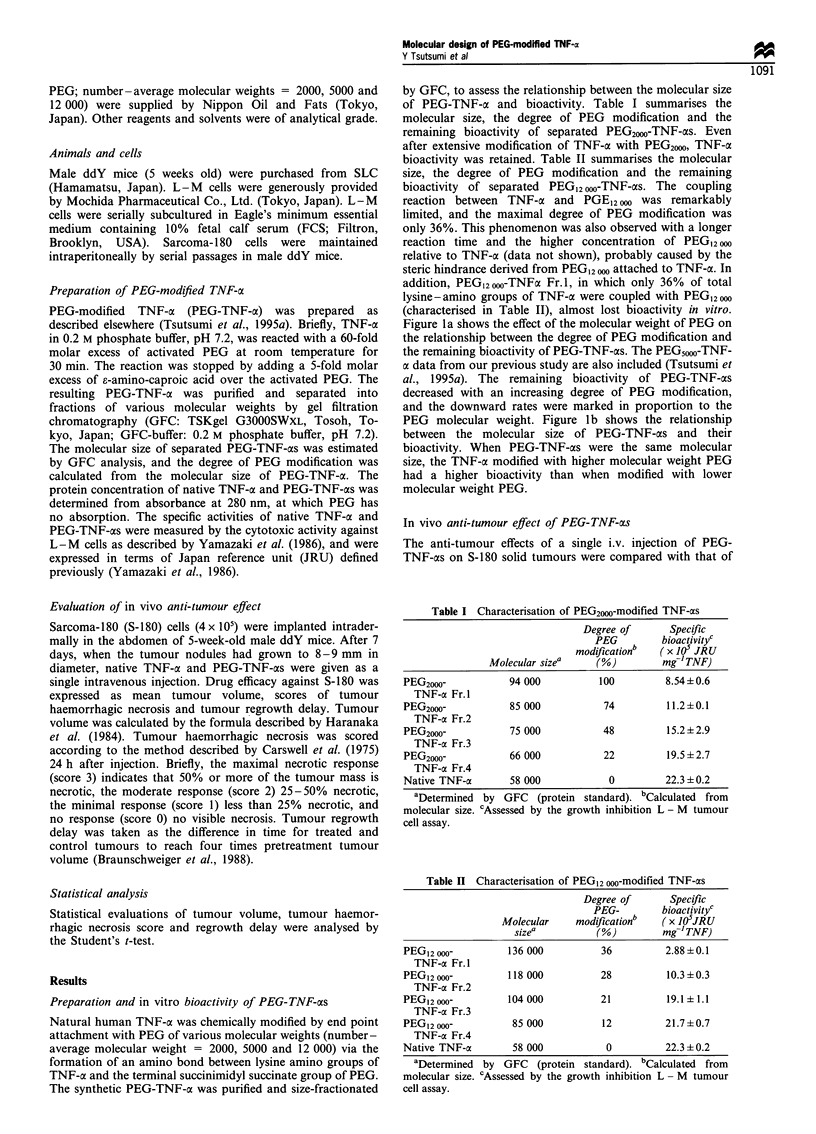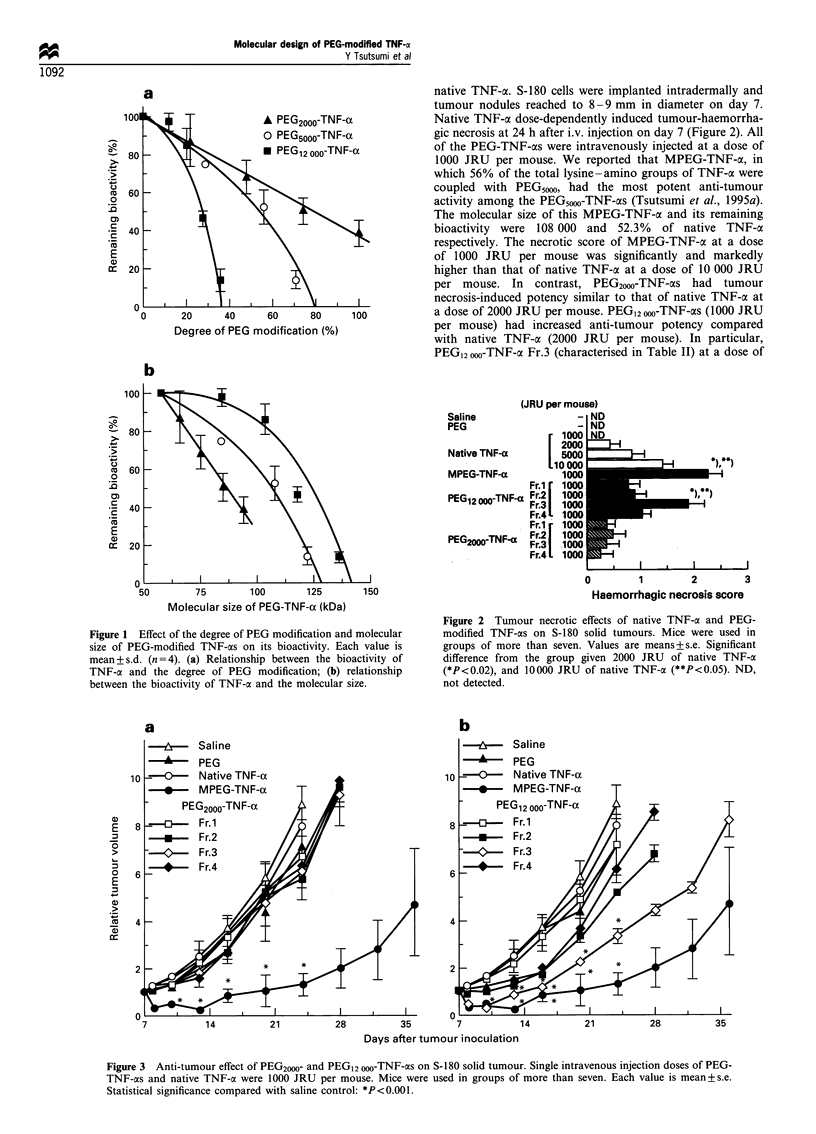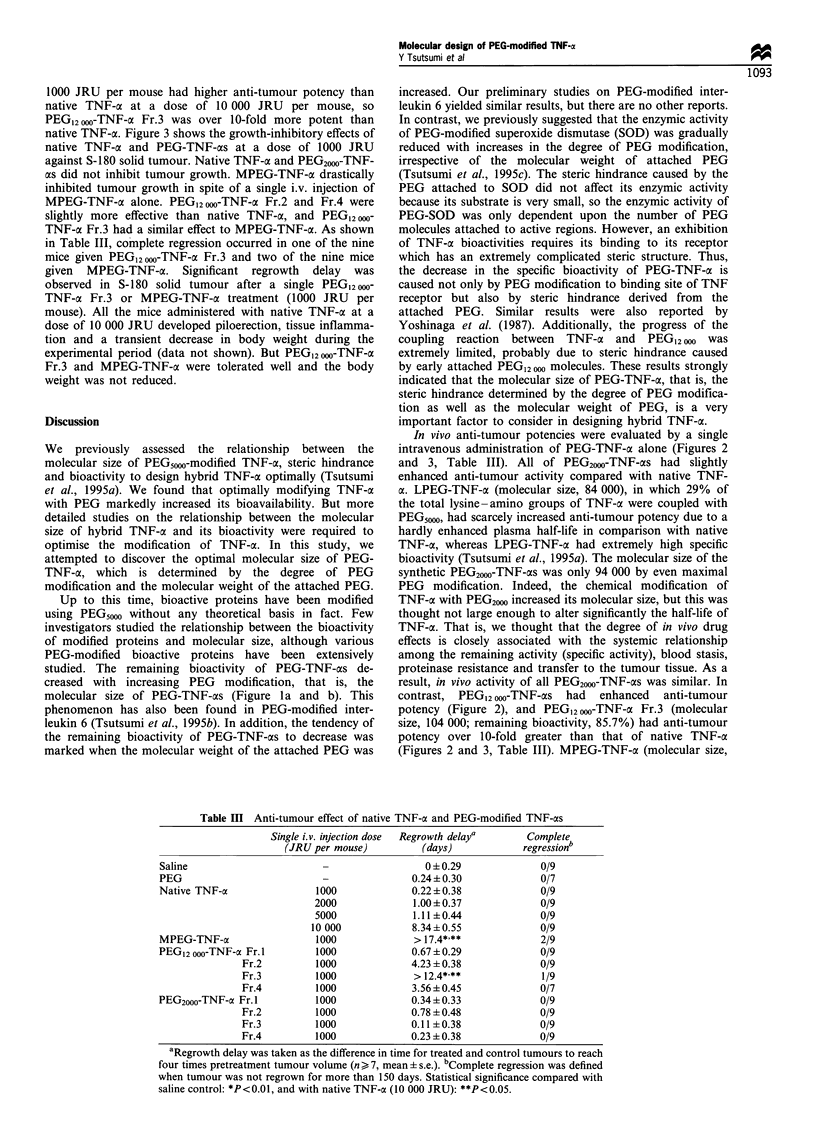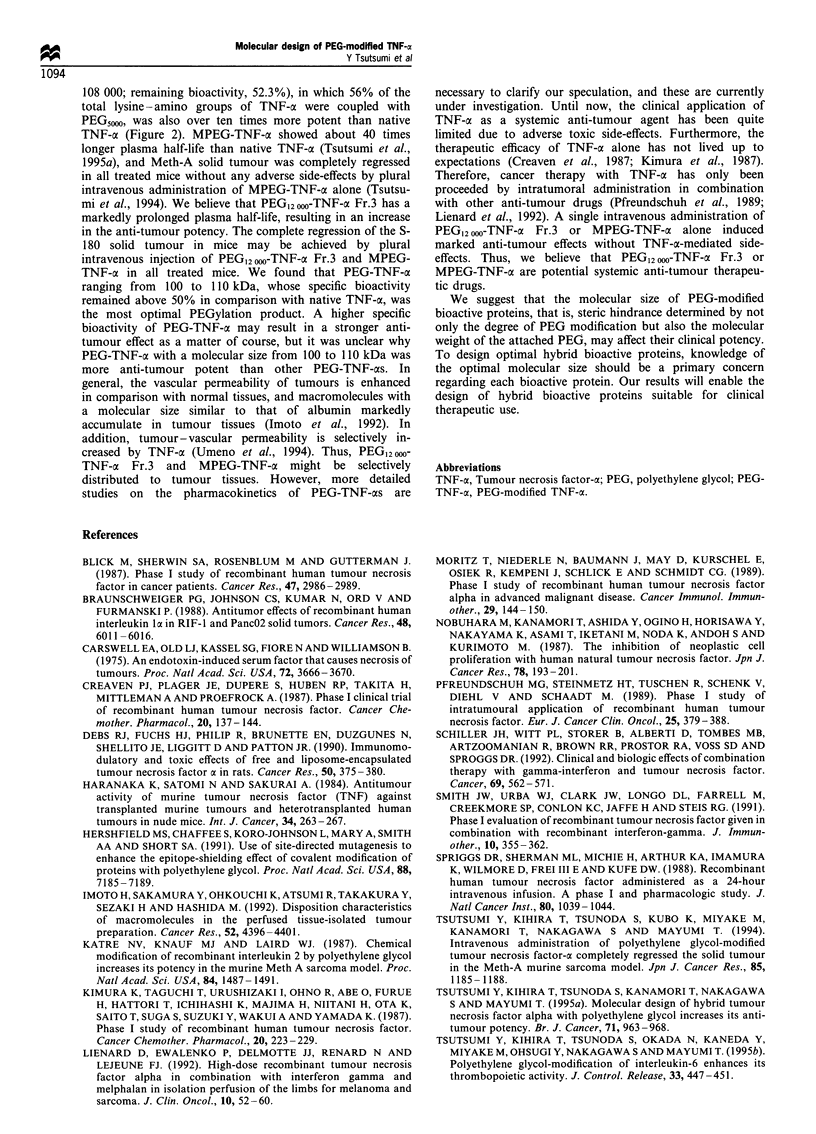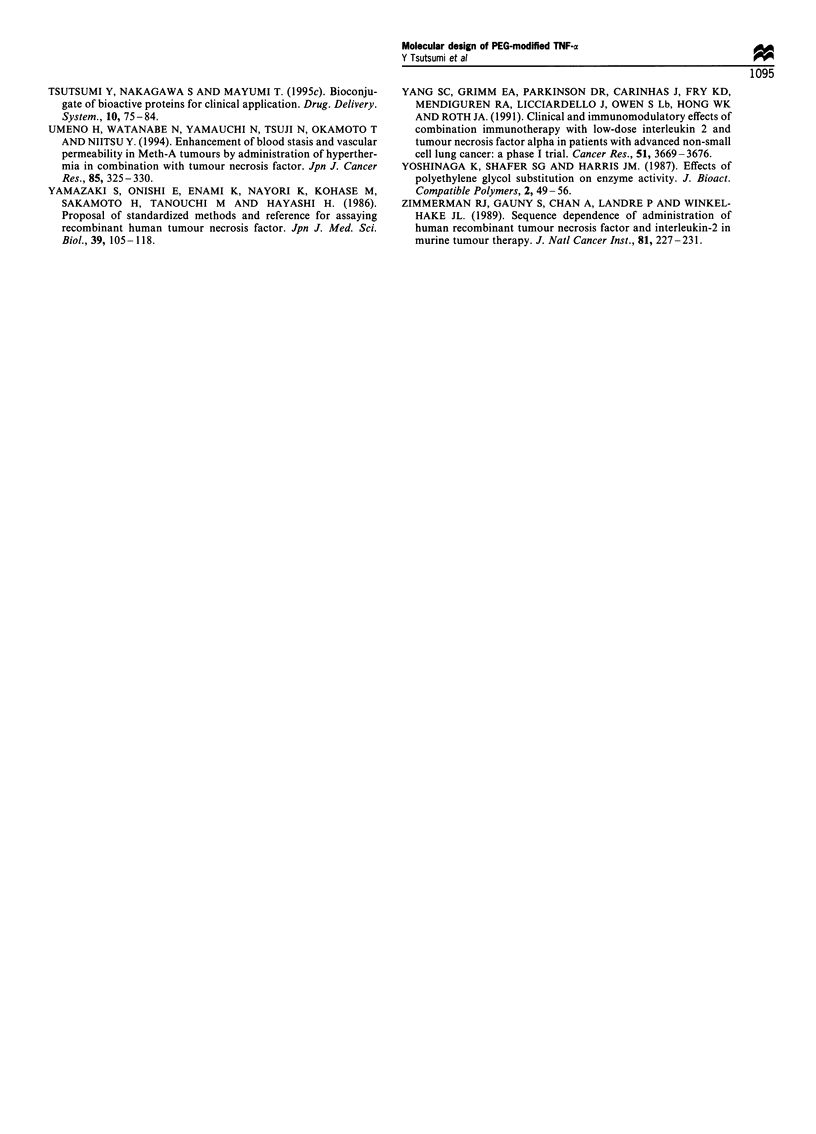# Molecular design of hybrid tumour necrosis factor-alpha. II: The molecular size of polyethylene glycol-modified tumour necrosis factor-alpha affects its anti-tumour potency.

**DOI:** 10.1038/bjc.1996.495

**Published:** 1996-10

**Authors:** Y. Tsutsumi, S. Tsunoda, H. Kamada, T. Kihira, S. Nakagawa, Y. Kaneda, T. Kanamori, T. Mayumi

**Affiliations:** Faculty and Graduate School of Pharmaceutical Sciences, Osaka University, Japan.

## Abstract

To design hybrid tumour necrosis factor-alpha (TNF-alpha) applicable to systemic anti-tumour therapeutic use, we assessed the relationships among the molecular size of hybrid TNF-alpha, in vitro bioactivity and in vivo anti-tumour potency. Natural human TNF-alpha was covalently modified with polyethylene glycol (PEG) of various number-average molecular weights (Mn = 2000, 5000, 12,000). The in vitro bioactivity of PEG-modified TNF-alpha s decreased with an increase in the degree of PEG modification, irrespective of the molecular weight of PEG. This decrease in the specific bioactivity markedly increased with an increase in the molecular weight of the attached PEG. The in vivo anti-tumour effects of the hybrid TNF-alpha s with a molecular size from 100 to 110 kDa, which had more than 50% of specific bioactivity of native TNF-alpha, were significantly superior to other PEG-TNF-alpha s. These hybrid TNF-alpha s showed over ten times greater anti-tumour effects than native TNF-alpha. Thus, the molecular size, which was determined by the degree of PEG modification and PEG molecular weight, influences the specific activity and anti-tumour effects of hybrid TNF-alpha.


British Journal of Cancer (1996) 74, 1090-1095
© 1996 Stockton Press All rights reserved 0007-0920/96 $12.00

### Molecular design of hybrid tumour necrosis factor-α II: the molecular size of polyethylene glycol-modified tumour necrosis factor-α affects its anti-tumour potency

Y Tsutsumi[1], S Tsunoda[1], H Kamada[1], T Kihira[1], S Nakagawa[1], Y Kaneda[1], T Kanamori[2] and T Mayumi[1]

[1]*Faculty and Graduate School of Pharmaceutical Sciences, Osaka University, 1-6 Yamadaoka, Suita, Osaka 565, Japan;* [2]*Research Laboratories for Cell Science, Mochida Pharmaceutical Co., Ltd., 1-1 Kamiya, Kita-ku, Tokyo 115, Japan.*

**Summary** To design hybrid tumour necrosis factor-α (TNF-α) applicable to systemic anti-tumour therapeutic use, we assessed the relationships among the molecular size of hybrid TNF-α, *in vitro* bioactivity and *in vivo* anti-tumour potency. Natural human TNF-α was covalently modified with polyethylene glycol (PEG) of various number-average molecular weights ($M_n$=2000, 5000, 12 000). The *in vitro* bioactivity of PEG-modified TNF-αs decreased with an increase in the degree of PEG modification, irrespective of the molecular weight of PEG. This decrease in the specific bioactivity markedly increased with an increase in the molecular weight of the attached PEG. The *in vivo* anti-tumour effects of the hybrid TNF-αs with a molecular size from 100 to 110 kDa, which had more than 50% of specific bioactivity of native TNF-α, were significantly superior to other PEG-TNF-αs. These hybrid TNF-αs showed over ten times greater anti-tumour effects than native TNF-α. Thus, the molecular size, which was determined by the degree of PEG modification and PEG molecular weight, influences the specific activity and anti-tumour effects of hybrid TNF-α.

**Keywords:** tumour necrosis factor-α; polyethylene glycol; molecular design; degree of modification; molecular size; molecular weight

Tumour necrosis factor-α (TNF-α), an anti-tumour cytokine produced by activated macrophages, has numerous biological effects, such as direct cytotoxicity against tumour cells, activation of immune anti-tumour response and selective impairment of tumour blood vessels (Nobuhara *et al.*, 1987; Debs *et al.*, 1990). Although TNF-α has been considered as a novel anti-tumour drug, its therapeutic application as a single and systemic anti-tumour agent is limited by the toxic side-effects revealed by clinical trials (Blick *et al.*, 1987; Spriggs *et al.*, 1988; Moritz *et al.*, 1989). TNF-α has therefore been therapeutically assessed in combination with other anti-tumour cytokines to treat several tumours, and synergistic effects have been identified (Zimmerman *et al.*, 1989). More recently, evidence has been accumulated that the adverse side-effects of TNF-α are substantially enhanced by its combination with interferon-γ and interleukin-2, when systemically administered (Smith *et al.*, 1991; Yang *et al.*, 1991; Schiller *et al.*, 1992). Nowadays, the clinical application of TNF-α is limited to intratumoral administration, and its clinical consequence is unfavourable (Pfreundschuh *et al.*, 1989; Lienard *et al.*, 1992).

Chemical modification of bioactive proteins with polyethylene glycol (PEG) increases their molecular size and steric hindrance, both of which are derived from PEG attached to bioactive proteins, resulting in augmented plasma half-lives and stability (Katre *et al.*, 1987; Hershfield *et al.*, 1991). These PEG-modified bioactive proteins have increased therapeutic potency, so PEGylation enables the therapeutic dose and frequency to be decreased. Thus, it seems that PEGylation of biological proteins is one of the most useful strategies to increase markedly their therapeutic efficacy and effectively reduce their toxic side-effects. However, clinical application of PEG-modified bioactive proteins has been limited as yet. This limitation of clinical application is due to the following reasons: 1) the increase in the molecular size of bioactive proteins by PEGylation restricts their distribution from blood to target tissues as well as increases their plasma half-lives; 2) steric hindrance not only protects bioactive proteins from attack by various proteinases, but also inhibits their receptor binding. We previously assessed the relationship between the molecular size of PEG-modified TNF-α, steric hindrance and bioactivity to design a hybrid TNF-α applicable to clinical use (Tsutsumi *et al.*, 1995*a*). As a result, the optimal modification of TNF-α with PEG ($PEG_{5000}$; number-average molecular weight, 5000) markedly and selectively increased its anti-tumour potency and effectively reduced its systemic toxic side-effects (Tsutsumi *et al.*, 1995*a*). In particular, MPEG-TNF-α, in which 56% of the lysine amino groups of TNF-α were coupled with $PEG_{5000}$, had more than 10-fold greater anti-tumour potency than native TNF-α, and several intravenous administrations of MPEG-TNF-α alone completely regressed Meth-A solid tumours in all treated mice without any TNF-α-mediated side-effects (Tsutsumi *et al.*, 1994). However, as pointed out in the previous report (Tsutsumi *et al.*, 1995*a*), more detailed studies of the relationship between the molecular size of hybrid TNF-α and its *in vivo* anti-tumour potency are necessary to design a more anti-tumour active hybrid TNF-α. We indicated that the molecular size of hybrid TNF-α, which is determined by the steric hindrance resulting from the molecular weight of attached PEG and the degree of PEG modification, may influence its specific activity and *in vivo* anti-tumour activity.

Here, we attempted to optimise the PEGylation of TNF-α to increase its anti-tumour potency. Hybrid TNF-αs were synthesised with PEG of various molecular weights and separated into various molecular size fractions to study the relationship between the molecular size of hybrid TNF-α, *in vitro* bioactivity and *in vivo* anti-tumour potency. This study provides the information necessary to design a hybrid TNF-α optimally suitable for therapeutic use.

### Materials and methods

### *Materials*

Natural human TNF-α was kindly supplied by Hayashibara Biological Laboratories Inc. (Okayama, Japan). *N*-succinimidyl succinate monomethoxy polyethylene glycol (activated

Correspondence: T Mayumi
Received 10 October 1995; revised 15 April 1996; accepted 24 April 1996

PEG; number-average molecular weights = 2000, 5000 and 12 000) were supplied by Nippon Oil and Fats (Tokyo, Japan). Other reagents and solvents were of analytical grade.

*Animals and cells*

Male ddY mice (5 weeks old) were purchased from SLC (Hamamatsu, Japan). L-M cells were generously provided by Mochida Pharmaceutical Co., Ltd. (Tokyo, Japan). L-M cells were serially subcultured in Eagle's minimum essential medium containing 10% fetal calf serum (FCS; Filtron, Brooklyn, USA). Sarcoma-180 cells were maintained intraperitoneally by serial passages in male ddY mice.

*Preparation of PEG-modified TNF-α*

PEG-modified TNF-α (PEG-TNF-α) was prepared as described elsewhere (Tsutsumi *et al.*, 1995*a*). Briefly, TNF-α in 0.2 M phosphate buffer, pH 7.2, was reacted with a 60-fold molar excess of activated PEG at room temperature for 30 min. The reaction was stopped by adding a 5-fold molar excess of ε-amino-caproic acid over the activated PEG. The resulting PEG-TNF-α was purified and separated into fractions of various molecular weights by gel filtration chromatography (GFC: TSKgel G3000SWXL, Tosoh, Tokyo, Japan; GFC-buffer: 0.2 M phosphate buffer, pH 7.2). The molecular size of separated PEG-TNF-αs was estimated by GFC analysis, and the degree of PEG modification was calculated from the molecular size of PEG-TNF-α. The protein concentration of native TNF-α and PEG-TNF-αs was determined from absorbance at 280 nm, at which PEG has no absorption. The specific activities of native TNF-α and PEG-TNF-αs were measured by the cytotoxic activity against L-M cells as described by Yamazaki *et al.* (1986), and were expressed in terms of Japan reference unit (JRU) defined previously (Yamazaki *et al.*, 1986).

*Evaluation of* in vivo *anti-tumour effect*

Sarcoma-180 (S-180) cells ($4 \times 10^5$) were implanted intradermally in the abdomen of 5-week-old male ddY mice. After 7 days, when the tumour nodules had grown to 8-9 mm in diameter, native TNF-α and PEG-TNF-αs were given as a single intravenous injection. Drug efficacy against S-180 was expressed as mean tumour volume, scores of tumour haemorrhagic necrosis and tumour regrowth delay. Tumour volume was calculated by the formula described by Haranaka *et al.* (1984). Tumour haemorrhagic necrosis was scored according to the method described by Carswell *et al.* (1975) 24 h after injection. Briefly, the maximal necrotic response (score 3) indicates that 50% or more of the tumour mass is necrotic, the moderate response (score 2) 25-50% necrotic, the minimal response (score 1) less than 25% necrotic, and no response (score 0) no visible necrosis. Tumour regrowth delay was taken as the difference in time for treated and control tumours to reach four times pretreatment tumour volume (Braunschweiger *et al.*, 1988).

*Statistical analysis*

Statistical evaluations of tumour volume, tumour haemorrhagic necrosis score and regrowth delay were analysed by the Student's *t*-test.

### Results

*Preparation and* in vitro *bioactivity of PEG-TNF-*αs

Natural human TNF-α was chemically modified by end point attachment with PEG of various molecular weights (number-average molecular weight = 2000, 5000 and 12 000) via the formation of an amino bond between lysine amino groups of TNF-α and the terminal succinimidyl succinate group of PEG. The synthetic PEG-TNF-α was purified and size-fractionated by GFC, to assess the relationship between the molecular size of PEG-TNF-α and bioactivity. Table I summarises the molecular size, the degree of PEG modification and the remaining bioactivity of separated $PEG_{2000}$-TNF-αs. Even after extensive modification of TNF-α with $PEG_{2000}$, TNF-α bioactivity was retained. Table II summarises the molecular size, the degree of PEG modification and the remaining bioactivity of separated $PEG_{12\,000}$-TNF-αs. The coupling reaction between TNF-α and $PGE_{12\,000}$ was remarkably limited, and the maximal degree of PEG modification was only 36%. This phenomenon was also observed with a longer reaction time and the higher concentration of $PEG_{12\,000}$ relative to TNF-α (data not shown), probably caused by the steric hindrance derived from $PEG_{12\,000}$ attached to TNF-α. In addition, $PEG_{12\,000}$-TNFα Fr.1, in which only 36% of total lysine-amino groups of TNF-α were coupled with $PEG_{12\,000}$ (characterised in Table II), almost lost bioactivity *in vitro*. Figure 1a shows the effect of the molecular weight of PEG on the relationship between the degree of PEG modification and the remaining bioactivity of PEG-TNF-αs. The $PEG_{5000}$-TNF-α data from our previous study are also included (Tsutsumi *et al.*, 1995*a*). The remaining bioactivity of PEG-TNF-αs decreased with an increasing degree of PEG modification, and the downward rates were marked in proportion to the PEG molecular weight. Figure 1b shows the relationship between the molecular size of PEG-TNF-αs and their bioactivity. When PEG-TNF-αs were the same molecular size, the TNF-α modified with higher molecular weight PEG had a higher bioactivity than when modified with lower molecular weight PEG.

In vivo *anti-tumour effect of PEG-TNF-αs*

The anti-tumour effects of a single i.v. injection of PEG-TNF-αs on S-180 solid tumours were compared with that of

**Table I** Characterisation of $PEG_{2000}$-modified TNF-αs

| | *Molecular size*[a] | *Degree of PEG modification*[b] *(%)* | *Specific bioactivity*[c] *($\times 10^5$ JRU $mg^{-1}$TNF)* |
|---|---|---|---|
| $PEG_{2000}$-TNF-α Fr.1 | 94 000 | 100 | $8.54 \pm 0.6$ |
| $PEG_{2000}$-TNF-α Fr.2 | 85 000 | 74 | $11.2 \pm 0.1$ |
| $PEG_{2000}$-TNF-α Fr.3 | 75 000 | 48 | $15.2 \pm 2.9$ |
| $PEG_{2000}$-TNF-α Fr.4 | 66 000 | 22 | $19.5 \pm 2.7$ |
| Native TNF-α | 58 000 | 0 | $22.3 \pm 0.2$ |

[a]Determined by GFC (protein standard). [b]Calculated from molecular size. [c]Assessed by the growth inhibition L-M tumour cell assay.

**Table II** Characterisation of $PEG_{12\,000}$-modified TNF-αs

| | *Molecular size*[a] | *Degree of PEG-modification*[b] *(%)* | *Specific bioactivity*[c] *($\times 10^5$JRU $mg^{-1}$TNF)* |
|---|---|---|---|
| $PEG_{12\,000}$-TNF-α Fr.1 | 136 000 | 36 | $2.88 \pm 0.1$ |
| $PEG_{12\,000}$-TNF-α Fr.2 | 118 000 | 28 | $10.3 \pm 0.3$ |
| $PEG_{12\,000}$-TNF-α Fr.3 | 104 000 | 21 | $19.1 \pm 1.1$ |
| $PEG_{12\,000}$-TNF-α Fr.4 | 85 000 | 12 | $21.7 \pm 0.7$ |
| Native TNF-α | 58 000 | 0 | $22.3 \pm 0.2$ |

[a]Determined by GFC (protein standard). [b]Calculated from molecular size. [c]Assessed by the growth inhibition L-M tumour cell assay.

**Figure 1** Effect of the degree of PEG modification and molecular size of PEG-modified TNF-αs on its bioactivity. Each value is mean ± s.d. ($n = 4$). (**a**) Relationship between the bioactivity of TNF-α and the degree of PEG modification; (**b**) relationship between the bioactivity of TNF-α and the molecular size.

native TNF-α. S-180 cells were implanted intradermally and tumour nodules reached to 8–9 mm in diameter on day 7. Native TNF-α dose-dependently induced tumour-haemorrhagic necrosis at 24 h after i.v. injection on day 7 (Figure 2). All of the PEG-TNF-αs were intravenously injected at a dose of 1000 JRU per mouse. We reported that MPEG-TNF-α, in which 56% of the total lysine–amino groups of TNF-α were coupled with $PEG_{5000}$, had the most potent anti-tumour activity among the $PEG_{5000}$-TNF-αs (Tsutsumi *et al.*, 1995*a*). The molecular size of this MPEG-TNF-α and its remaining bioactivity were 108 000 and 52.3% of native TNF-α respectively. The necrotic score of MPEG-TNF-α at a dose of 1000 JRU per mouse was significantly and markedly higher than that of native TNF-α at a dose of 10 000 JRU per mouse. In contrast, $PEG_{2000}$-TNF-αs had tumour necrosis-induced potency similar to that of native TNF-α at a dose of 2000 JRU per mouse. $PEG_{12\,000}$-TNF-αs (1000 JRU per mouse) had increased anti-tumour potency compared with native TNF-α (2000 JRU per mouse). In particular, $PEG_{12\,000}$-TNF-α Fr.3 (characterised in Table II) at a dose of

**Figure 2** Tumour necrotic effects of native TNF-α and PEG-modified TNF-αs on S-180 solid tumours. Mice were used in groups of more than seven. Values are means ± s.e. Significant difference from the group given 2000 JRU of native TNF-α ($^*P < 0.02$), and 10 000 JRU of native TNF-α ($^{**}P < 0.05$). ND, not detected.

**Figure 3** Anti-tumour effect of $PEG_{2000}$- and $PEG_{12\,000}$-TNF-αs on S-180 solid tumour. Single intravenous injection doses of PEG-TNF-αs and native TNF-α were 1000 JRU per mouse. Mice were used in groups of more than seven. Each value is mean ± s.e. Statistical significance compared with saline control: $^*P < 0.001$.

1000 JRU per mouse had higher anti-tumour potency than native TNF-α at a dose of 10 000 JRU per mouse, so $PEG_{12\,000}$-TNF-α Fr.3 was over 10-fold more potent than native TNF-α. Figure 3 shows the growth-inhibitory effects of native TNF-α and PEG-TNF-αs at a dose of 1000 JRU against S-180 solid tumour. Native TNF-α and $PEG_{2000}$-TNF-αs did not inhibit tumour growth. MPEG-TNF-α drastically inhibited tumour growth in spite of a single i.v. injection of MPEG-TNF-α alone. $PEG_{12\,000}$-TNF-α Fr.2 and Fr.4 were slightly more effective than native TNF-α, and $PEG_{12\,000}$-TNF-α Fr.3 had a similar effect to MPEG-TNF-α. As shown in Table III, complete regression occurred in one of the nine mice given $PEG_{12\,000}$-TNF-α Fr.3 and two of the nine mice given MPEG-TNF-α. Significant regrowth delay was observed in S-180 solid tumour after a single $PEG_{12\,000}$-TNF-α Fr.3 or MPEG-TNF-α treatment (1000 JRU per mouse). All the mice administered with native TNF-α at a dose of 10 000 JRU developed piloerection, tissue inflammation and a transient decrease in body weight during the experimental period (data not shown). But $PEG_{12\,000}$-TNF-α Fr.3 and MPEG-TNF-α were tolerated well and the body weight was not reduced.

### Discussion

We previously assessed the relationship between the molecular size of $PEG_{5000}$-modified TNF-α, steric hindrance and bioactivity to design hybrid TNF-α optimally (Tsutsumi *et al.*, 1995*a*). We found that optimally modifying TNF-α with PEG markedly increased its bioavailability. But more detailed studies on the relationship between the molecular size of hybrid TNF-α and its bioactivity were required to optimise the modification of TNF-α. In this study, we attempted to discover the optimal molecular size of PEG-TNF-α, which is determined by the degree of PEG modification and the molecular weight of the attached PEG.

Up to this time, bioactive proteins have been modified using $PEG_{5000}$ without any theoretical basis in fact. Few investigators studied the relationship between the bioactivity of modified proteins and molecular size, although various PEG-modified bioactive proteins have been extensively studied. The remaining bioactivity of PEG-TNF-αs decreased with increasing PEG modification, that is, the molecular size of PEG-TNF-αs (Figure 1a and b). This phenomenon has also been found in PEG-modified interleukin 6 (Tsutsumi *et al.*, 1995*b*). In addition, the tendency of the remaining bioactivity of PEG-TNF-αs to decrease was marked when the molecular weight of the attached PEG was increased. Our preliminary studies on PEG-modified interleukin 6 yielded similar results, but there are no other reports. In contrast, we previously suggested that the enzymic activity of PEG-modified superoxide dismutase (SOD) was gradually reduced with increases in the degree of PEG modification, irrespective of the molecular weight of attached PEG (Tsutsumi *et al.*, 1995*c*). The steric hindrance caused by the PEG attached to SOD did not affect its enzymic activity because its substrate is very small, so the enzymic activity of PEG-SOD was only dependent upon the number of PEG molecules attached to active regions. However, an exhibition of TNF-α bioactivities requires its binding to its receptor which has an extremely complicated steric structure. Thus, the decrease in the specific bioactivity of PEG-TNF-α is caused not only by PEG modification to binding site of TNF receptor but also by steric hindrance derived from the attached PEG. Similar results were also reported by Yoshinaga *et al.* (1987). Additionally, the progress of the coupling reaction between TNF-α and $PEG_{12\,000}$ was extremely limited, probably due to steric hindrance caused by early attached $PEG_{12\,000}$ molecules. These results strongly indicated that the molecular size of PEG-TNF-α, that is, the steric hindrance determined by the degree of PEG modification as well as the molecular weight of PEG, is a very important factor to consider in designing hybrid TNF-α.

*In vivo* anti-tumour potencies were evaluated by a single intravenous administration of PEG-TNF-α alone (Figures 2 and 3, Table III). All of $PEG_{2000}$-TNF-αs had slightly enhanced anti-tumour activity compared with native TNF-α. LPEG-TNF-α (molecular size, 84 000), in which 29% of the total lysine–amino groups of TNF-α were coupled with $PEG_{5000}$, had scarcely increased anti-tumour potency due to a hardly enhanced plasma half-life in comparison with native TNF-α, whereas LPEG-TNF-α had extremely high specific bioactivity (Tsutsumi *et al.*, 1995*a*). The molecular size of the synthetic $PEG_{2000}$-TNF-αs was only 94 000 by even maximal PEG modification. Indeed, the chemical modification of TNF-α with $PEG_{2000}$ increased its molecular size, but this was thought not large enough to alter significantly the half-life of TNF-α. That is, we thought that the degree of *in vivo* drug effects is closely associated with the systemic relationship among the remaining activity (specific activity), blood stasis, proteinase resistance and transfer to the tumour tissue. As a result, *in vivo* activity of all $PEG_{2000}$-TNF-αs was similar. In contrast, $PEG_{12\,000}$-TNF-αs had enhanced anti-tumour potency (Figure 2), and $PEG_{12\,000}$-TNF-α Fr.3 (molecular size, 104 000; remaining bioactivity, 85.7%) had anti-tumour potency over 10-fold greater than that of native TNF-α (Figures 2 and 3, Table III). MPEG-TNF-α (molecular size,

**Table III** Anti-tumour effect of native TNF-α and PEG-modified TNF-αs

| | *Single i.v. injection dose (JRU per mouse)* | *Regrowth delay*[a] *(days)* | *Complete regression*[b] |
|---|---|---|---|
| Saline | – | $0 \pm 0.29$ | 0/9 |
| PEG | – | $0.24 \pm 0.30$ | 0/7 |
| Native TNF-α | 1000 | $0.22 \pm 0.38$ | 0/9 |
| | 2000 | $1.00 \pm 0.37$ | 0/9 |
| | 5000 | $1.11 \pm 0.44$ | 0/9 |
| | 10 000 | $8.34 \pm 0.55$ | 0/9 |
| MPEG-TNF-α | 1000 | $>17.4^{*,**}$ | 2/9 |
| $PEG_{12\,000}$-TNF-α Fr.1 | 1000 | $0.67 \pm 0.29$ | 0/9 |
| Fr.2 | 1000 | $4.23 \pm 0.38$ | 0/9 |
| Fr.3 | 1000 | $>12.4^{*,**}$ | 1/9 |
| Fr.4 | 1000 | $3.56 \pm 0.45$ | 0/7 |
| $PEG_{2000}$-TNF-α Fr.1 | 1000 | $0.34 \pm 0.33$ | 0/9 |
| Fr.2 | 1000 | $0.78 \pm 0.48$ | 0/9 |
| Fr.3 | 1000 | $0.11 \pm 0.38$ | 0/9 |
| Fr.4 | 1000 | $0.23 \pm 0.38$ | 0/9 |

[a]Regrowth delay was taken as the difference in time for treated and control tumours to reach four times pretreatment tumour volume ($n \geq 7$, mean ± s.e.). [b]Complete regression was defined when tumour was not regrown for more than 150 days. Statistical significance compared with saline control: $^{*}P<0.01$, and with native TNF-α (10 000 JRU): $^{**}P<0.05$.

108 000; remaining bioactivity, 52.3%), in which 56% of the total lysine-amino groups of TNF-α were coupled with $PEG_{5000}$, was also over ten times more potent than native TNF-α (Figure 2). MPEG-TNF-α showed about 40 times longer plasma half-life than native TNF-α (Tsutsumi *et al.*, 1995*a*), and Meth-A solid tumour was completely regressed in all treated mice without any adverse side-effects by plural intravenous administration of MPEG-TNF-α alone (Tsutsumi *et al.*, 1994). We believe that $PEG_{12\,000}$-TNF-α Fr.3 has a markedly prolonged plasma half-life, resulting in an increase in the anti-tumour potency. The complete regression of the S-180 solid tumour in mice may be achieved by plural intravenous injection of $PEG_{12\,000}$-TNF-α Fr.3 and MPEG-TNF-α in all treated mice. We found that PEG-TNF-α ranging from 100 to 110 kDa, whose specific bioactivity remained above 50% in comparison with native TNF-α, was the most optimal PEGylation product. A higher specific bioactivity of PEG-TNF-α may result in a stronger anti-tumour effect as a matter of course, but it was unclear why PEG-TNF-α with a molecular size from 100 to 110 kDa was more anti-tumour potent than other PEG-TNF-αs. In general, the vascular permeability of tumours is enhanced in comparison with normal tissues, and macromolecules with a molecular size similar to that of albumin markedly accumulate in tumour tissues (Imoto *et al.*, 1992). In addition, tumour-vascular permeability is selectively increased by TNF-α (Umeno *et al.*, 1994). Thus, $PEG_{12\,000}$-TNF-α Fr.3 and MPEG-TNF-α might be selectively distributed to tumour tissues. However, more detailed studies on the pharmacokinetics of PEG-TNF-αs are necessary to clarify our speculation, and these are currently under investigation. Until now, the clinical application of TNF-α as a systemic anti-tumour agent has been quite limited due to adverse toxic side-effects. Furthermore, the therapeutic efficacy of TNF-α alone has not lived up to expectations (Creaven *et al.*, 1987; Kimura *et al.*, 1987). Therefore, cancer therapy with TNF-α has only been proceeded by intratumoral administration in combination with other anti-tumour drugs (Pfreundschuh *et al.*, 1989; Lienard *et al.*, 1992). A single intravenous administration of $PEG_{12\,000}$-TNF-α Fr.3 or MPEG-TNF-α alone induced marked anti-tumour effects without TNF-α-mediated side-effects. Thus, we believe that $PEG_{12\,000}$-TNF-α Fr.3 or MPEG-TNF-α are potential systemic anti-tumour therapeutic drugs.

We suggest that the molecular size of PEG-modified bioactive proteins, that is, steric hindrance determined by not only the degree of PEG modification but also the molecular weight of the attached PEG, may affect their clinical potency. To design optimal hybrid bioactive proteins, knowledge of the optimal molecular size should be a primary concern regarding each bioactive protein. Our results will enable the design of hybrid bioactive proteins suitable for clinical therapeutic use.

**Abbreviations**

TNF-α, Tumour necrosis factor-α; PEG, polyethylene glycol; PEG-TNF-α, PEG-modified TNF-α.

### References

BLICK M, SHERWIN SA, ROSENBLUM M AND GUTTERMAN J. (1987). Phase I study of recombinant human tumour necrosis factor in cancer patients. *Cancer Res.*, **47**, 2986-2989.

BRAUNSCHWEIGER PG, JOHNSON CS, KUMAR N, ORD V AND FURMANSKI P. (1988). Antitumor effects of recombinant human interleukin 1α in RIF-1 and Panc02 solid tumors. *Cancer Res.*, **48**, 6011-6016.

CARSWELL EA, OLD LJ, KASSEL SG, FIORE N AND WILLIAMSON B. (1975). An endotoxin-induced serum factor that causes necrosis of tumours. *Proc. Natl Acad. Sci. USA*, **72**, 3666-3670.

CREAVEN PJ, PLAGER JE, DUPERE S, HUBEN RP, TAKITA H, MITTLEMAN A AND PROEFROCK A. (1987). Phase I clinical trial of recombinant human tumour necrosis factor. *Cancer Chemother. Pharmacol.*, **20**, 137-144.

DEBS RJ, FUCHS HJ, PHILIP R, BRUNETTE EN, DUZGUNES N, SHELLITO JE, LIGGITT D AND PATTON JR. (1990). Immunomodulatory and toxic effects of free and liposome-encapsulated tumour necrosis factor α in rats. *Cancer Res.*, **50**, 375-380.

HARANAKA K, SATOMI N AND SAKURAI A. (1984). Antitumour activity of murine tumour necrosis factor (TNF) against transplanted murine tumours and heterotransplanted human tumours in nude mice. *Int. J. Cancer*, **34**, 263-267.

HERSHFIELD MS, CHAFFEE S, KORO-JOHNSON L, MARY A, SMITH AA AND SHORT SA. (1991). Use of site-directed mutagenesis to enhance the epitope-shielding effect of covalent modification of proteins with polyethylene glycol. *Proc. Natl Acad. Sci. USA*, **88**, 7185-7189.

IMOTO H, SAKAMURA Y, OHKOUCHI K, ATSUMI R, TAKAKURA Y, SEZAKI H AND HASHIDA M. (1992). Disposition characteristics of macromolecules in the perfused tissue-isolated tumour preparation. *Cancer Res.*, **52**, 4396-4401.

KATRE NV, KNAUF MJ AND LAIRD WJ. (1987). Chemical modification of recombinant interleukin 2 by polyethylene glycol increases its potency in the murine Meth A sarcoma model. *Proc. Natl Acad. Sci. USA*, **84**, 1487-1491.

KIMURA K, TAGUCHI T, URUSHIZAKI I, OHNO R, ABE O, FURUE H, HATTORI T, ICHIHASHI K, MAJIMA H, NIITANI H, OTA K, SAITO T, SUGA S, SUZUKI Y, WAKUI A AND YAMADA K. (1987). Phase I study of recombinant human tumour necrosis factor. *Cancer Chemother. Pharmacol.*, **20**, 223-229.

LIENARD D, EWALENKO P, DELMOTTE JJ, RENARD N AND LEJEUNE FJ. (1992). High-dose recombinant tumour necrosis factor alpha in combination with interferon gamma and melphalan in isolation perfusion of the limbs for melanoma and sarcoma. *J. Clin. Oncol.*, **10**, 52-60.

MORITZ T, NIEDERLE N, BAUMANN J, MAY D, KURSCHEL E, OSIEK R, KEMPENI J, SCHLICK E AND SCHMIDT CG. (1989). Phase I study of recombinant human tumour necrosis factor alpha in advanced malignant disease. *Cancer Immunol. Immunother.*, **29**, 144-150.

NOBUHARA M, KANAMORI T, ASHIDA Y, OGINO H, HORISAWA Y, NAKAYAMA K, ASAMI T, IKETANI M, NODA K, ANDOH S AND KURIMOTO M. (1987). The inhibition of neoplastic cell proliferation with human natural tumour necrosis factor. *Jpn J. Cancer Res.*, **78**, 193-201.

PFREUNDSCHUH MG, STEINMETZ HT, TUSCHEN R, SCHENK V, DIEHL V AND SCHAADT M. (1989). Phase I study of intratumoural application of recombinant human tumour necrosis factor. *Eur. J. Cancer Clin. Oncol.*, **25**, 379-388.

SCHILLER JH, WITT PL, STORER B, ALBERTI D, TOMBES MB, ARTZOOMANIAN R, BROWN RR, PROSTOR RA, VOSS SD AND SPROGGS DR. (1992). Clinical and biologic effects of combination therapy with gamma-interferon and tumour necrosis factor. *Cancer*, **69**, 562-571.

SMITH JW, URBA WJ, CLARK JW, LONGO DL, FARRELL M, CREEKMORE SP, CONLON KC, JAFFE H AND STEIS RG. (1991). Phase I evaluation of recombinant tumour necrosis factor given in combination with recombinant interferon-gamma. *J. Immunother.*, **10**, 355-362.

SPRIGGS DR, SHERMAN ML, MICHIE H, ARTHUR KA, IMAMURA K, WILMORE D, FREI III E AND KUFE DW. (1988). Recombinant human tumour necrosis factor administered as a 24-hour intravenous infusion. A phase I and pharmacologic study. *J. Natl Cancer Inst.*, **80**, 1039-1044.

TSUTSUMI Y, KIHIRA T, TSUNODA S, KUBO K, MIYAKE M, KANAMORI T, NAKAGAWA S AND MAYUMI T. (1994). Intravenous administration of polyethylene glycol-modified tumour necrosis factor-α completely regressed the solid tumour in the Meth-A murine sarcoma model. *Jpn J. Cancer Res.*, **85**, 1185-1188.

TSUTSUMI Y, KIHIRA T, TSUNODA S, KANAMORI T, NAKAGAWA S AND MAYUMI T. (1995*a*). Molecular design of hybrid tumour necrosis factor alpha with polyethylene glycol increases its anti-tumour potency. *Br. J. Cancer*, **71**, 963-968.

TSUTSUMI Y, KIHIRA T, TSUNODA S, OKADA N, KANEDA Y, MIYAKE M, OHSUGI Y, NAKAGAWA S AND MAYUMI T. (1995*b*). Polyethylene glycol-modification of interleukin-6 enhances its thrombopoietic activity. *J. Control. Release*, **33**, 447-451.

TSUTSUMI Y, NAKAGAWA S AND MAYUMI T. (1995*c*). Bioconjugate of bioactive proteins for clinical application. *Drug. Delivery. System.*, **10**, 75–84.

UMENO H, WATANABE N, YAMAUCHI N, TSUJI N, OKAMOTO T AND NIITSU Y. (1994). Enhancement of blood stasis and vascular permeability in Meth-A tumours by administration of hyperthermia in combination with tumour necrosis factor. *Jpn J. Cancer Res.*, **85**, 325–330.

YAMAZAKI S, ONISHI E, ENAMI K, NAYORI K, KOHASE M, SAKAMOTO H, TANOUCHI M AND HAYASHI H. (1986). Proposal of standardized methods and reference for assaying recombinant human tumour necrosis factor. *Jpn J. Med. Sci. Biol.*, **39**, 105–118.

YANG SC, GRIMM EA, PARKINSON DR, CARINHAS J, FRY KD, MENDIGUREN RA, LICCIARDELLO J, OWEN S Lb, HONG WK AND ROTH JA. (1991). Clinical and immunomodulatory effects of combination immunotherapy with low-dose interleukin 2 and tumour necrosis factor alpha in patients with advanced non-small cell lung cancer: a phase I trial. *Cancer Res.*, **51**, 3669–3676.

YOSHINAGA K, SHAFER SG AND HARRIS JM. (1987). Effects of polyethylene glycol substitution on enzyme activity. *J. Bioact. Compatible Polymers*, **2**, 49–56.

ZIMMERMAN RJ, GAUNY S, CHAN A, LANDRE P AND WINKELHAKE JL. (1989). Sequence dependence of administration of human recombinant tumour necrosis factor and interleukin-2 in murine tumour therapy. *J. Natl Cancer Inst.*, **81**, 227–231.